# Current Practices and Perceived Effectiveness of Clinicians Regarding Polynucleotide Injection for Knee Osteoarthritis: A Survey-Based Evaluation

**DOI:** 10.3390/healthcare13020113

**Published:** 2025-01-09

**Authors:** Dagyeong Lee, Wan-ho Kim, Jeong Han Ha, Hyungjin Kim, Junbae Kim, Dong Wook Shin

**Affiliations:** 1 Department of Family Medicine, Hallym University Dongtan Sacred Heart Hospital, Hwaseong 18450, Republic of Korea; 2 Kim Wanho Orthopedic Clinic, Seoul 07702, Republic of Korea; aescular@naver.com; 3 Ha Jeong Han Orthopedic Clinic, Seoul 07055, Republic of Korea; jdihjh@nate.com; 4 Department of Medicine, Samsung Medical Center, Sungkyunkwan University School of Medicine, Seoul 06351, Republic of Korea; passiondoc@gmail.com; 5 Seoul NOW Hospital, Anyang 14058, Republic of Korea; 6 Department of Family Medicine, Supportive Care Center, Samsung Medical Center, Sungkyunkwan University School of Medicine, Seoul 06351, Republic of Korea

**Keywords:** polynucleotides, knee osteoarthritis, viscosupplementation

## Abstract

**Aims:** Intra-articular (IA) injection therapy, particularly IA hyaluronic acid (HA), is a common treatment for knee osteoarthritis, but it does have limitations. The injection of IA polynucleotide (PN) has emerged as an alternative, potentially offering superior clinical outcomes. This study investigates current practice patterns and the perceived effectiveness of PN among clinicians for treating knee osteoarthritis in the Republic of Korea. **Methods:** Based on a survey conducted among clinicians who use PN in clinical practice, we explored the current practices and assessed the perceived effectiveness of IA PN in treating knee osteoarthritis. **Results:** A total of 265 clinicians who used IA PN for knee osteoarthritis participated in the survey. Most clinicians (73.3%) used PN therapy for the treatment of chronic pain, with varying administration frequencies. In addition, 25.8% of clinicians used PN for the treatment of acute flare-ups. In cases of knee effusion, 55.5% of clinicians removed the effusion prior to administering PN. Clinicians rated PN as more effective than HA for both chronic pain and acute flare-ups, with higher scores for cushioning, anti-inflammatory effects, and delaying joint degeneration. The clinicians stated that patients expressed a higher satisfaction with IA PN compared with IA HA, noting improvement in joint smoothness, noise reduction, pain relief, and a reduction in heat sensation and swelling. **Conclusions:** The results of the present study highlight the extensive use and perceived benefits among clinicians of IA PN for knee osteoarthritis in the Republic of Korea. Further research is warranted to explore the effectiveness of PN in acute flare-ups and to validate these findings in broader populations.

## 1. Introduction

Intra-articular (IA) injection therapy plays a pivotal role in the treatment of knee osteoarthritis, particularly for patients who do not respond adequately to medication or have comorbidities that limit their use of medical treatments [1]. IA hyaluronic acid (HA) is a widely used treatment for knee osteoarthritis, with its clinical efficacy and safety supported in prior studies [2]. However, recent osteoarthritis treatment guidelines highlight the lack of generalized effectiveness as a limitation of IA HA treatment [1,3]. Furthermore, several patients have reported symptoms of pseudo-septic arthritis, such as painful swelling and redness, following IA HA use [4]. Similarly, while corticosteroids are effective in the treatment of acute inflammation, they are associated with cartilage damage, necessitating the development of alternative therapies that offer both safety and efficacy [5].

Over the past decade, IA polynucleotide (PN) has been proposed as an alternative to IA HA for viscosupplementation [6]. PNs are polymeric molecules composed of long-chain deoxyribonucleic acid fractions with high molecular weight, extracted from the testes and sperm of salmon [7]. The capability of PNs to bind large amounts of water provides viscoelasticity in the joint space [8], allowing its use as a mechanical supplement for the treatment of osteoarthritis. Moreover, PN exhibits anti-inflammatory properties and tissue repair potential by modulating inflammatory cytokines [9].

Previous studies have compared the clinical effectiveness of different viscosupplements for the treatment of painful knee osteoarthritis. A retrospective pilot study comparing classic HA, cross-linked HA, and PN demonstrated that PN more effectively reduced weight-bearing pain than classic and cross-linked HA [10]. IA PN has been suggested to provide comparable or superior clinical outcomes. For example, Vanelli et al. [6] and Giarratana et al. [11] found similar levels of pain reduction and improvement in clinical scores between IA PN and IA HA groups. Zazgyva et al. also reported significant pain reduction in two such groups [12].

Due to its proposed mechanisms and encouraging results from previous studies, PN is increasingly used for the treatment of knee osteoarthritis in the Republic of Korea [13]. There is limited research on several aspects of this drug. PN was originally approved under the reimbursement policy of the Korean National Insurance Health Service for chronic knee osteoarthritis with a regimen of five injections. The policy allows up to five injections over a six-month period. However, in actual clinical practice, the drug is often used in different ways, including in the acute phase of osteoarthritis. Moreover, the frequency of injections is determined by shared decision-making between surgeons and patients, based on patient satisfaction and treatment response. Despite its widespread use, there is a lack of studies examining the usage patterns of PN in real-world clinical practice. Additionally, there is no research on physicians’ perceptions of the effectiveness of PN compared to commonly used HA.

Therefore, in this survey study, we investigated the current practice and perceived effectiveness of PN in treating knee osteoarthritis among physicians in the Republic of Korea.

## 2. Methods

### 2.1. Survey Development Process

A comprehensive examination of existing research [6,10,11,12,13] facilitated the identification of current practices and perceptions of PN. Based on these findings, the survey was developed through face-to-face consultation with expert orthopedic surgeons. The further refinement of the survey was achieved through comparative discussions with rheumatologists. A pilot study involving five independent orthopedic surgeons was conducted to assess the clarity and validity of the survey. This process resulted in minor adjustments to question phrasing, with the aim of improving comprehension. It should be noted that the surgeons in question were not part of the main study cohort.

### 2.2. Survey Content

The questions were designed to collect information on current practices and the perceived effectiveness of IA PN for treatment of knee osteoarthritis. The survey explored clinicians’ usage patterns and compared the perceived effectiveness of IA PN with that of IA HA. The survey gathered outcomes and effectiveness from patients based on clinicians’ experiences.

### 2.3. Practice Patterns

The initial question sought information on clinician experience with PN usage. Respondents who chose ’less than 10 cases’ were excluded because this was considered a surrogate measure for the inclusion criterion of requiring relevant experience with PN.

The subsequent question pertained to the frequency of PN injections, asking whether clinicians administer PN in a single cycle (five injections over a six-month period) or periodically (every six months) throughout the course of the disease.

The survey also asked whether clinicians administer PN injections to patients with chronic knee osteoarthritis and, if so, how frequently in a cycle, with options ranging from a single to up to five injections. For acute flares in knee osteoarthritis patients, the survey asked about the use of PN injections, including the frequency of use per cycle or until symptoms improved.

In addition, the survey examined practices in cases of knee effusion based on the clinicians’ use of PN injections. Those that reported such use were asked whether they remove the effusion prior to administration, administer PN immediately without removal, or wait for a period of time before administering PN, either with or without effusion removal.

### 2.4. Perceived Effectiveness

Clinicians were asked if HA or PN injections are used for patients with K-L grades 1–4. The K-L grading system is widely used to assess the severity of knee osteoarthritis based on radiographic findings, ranging from Grade 0 (no OA features) to Grade 4 (severe OA with significant joint space narrowing and osteophyte formation). The clinicians rated the effectiveness of HA or PN injections for both chronic pain and the flare-up of acute knee osteoarthritis, using a 10-point scale where 0 indicates “ineffective” and 10 indicates “effective”. Clinicians rated the expected effects of HA and PN injections, including cushioning and lubrication, anti-inflammatory effects, and delaying joint degeneration. They also rated the effects reported by patients, including smoother movements, reduced noise, decreased pain, less heat, and reduced swelling.

The scores provided by the clinicians were based on their overall experience in treating knee osteoarthritis, rather than on assessments of individual cases. This approach was chosen to capture clinicians’ broad clinical expertise and general treatment strategies, reflecting their cumulative experience of treating patients over time. By focusing on overall experience, the study aimed to gain insights into the clinicians’ overall approach to managing knee osteoarthritis, rather than isolated assessments of individual cases.

### 2.5. Data Collection

The survey was distributed at the Korean Association of Orthopedic Surgeons and the Korean Knee Society meetings in Seoul, Republic of Korea, on 31 March 2024 and 17–18 May 2024. The survey was accessed via encrypted URLs on mobile devices, thereby ensuring that the responses were unique and that data security was maintained. All identifying information was anonymized. Informed consent forms were obtained, and the protocol was approved by Samsung Medical Center Institutional Review Board (SMC-2024-02-097).

### 2.6. Statistical Analysis

Categorical data are presented as proportions. First, frequency analysis was performed to understand the general characteristics of the study participants. Second, descriptive statistics and graphical representations were used to summarize the data. All statistical analyses were performed using R version 4.3.1 software (R Foundation for Statistical Computing, Vienna, Austria).

## 3. Results

### 3.1. Baseline Characteristics

**Figure 1:**
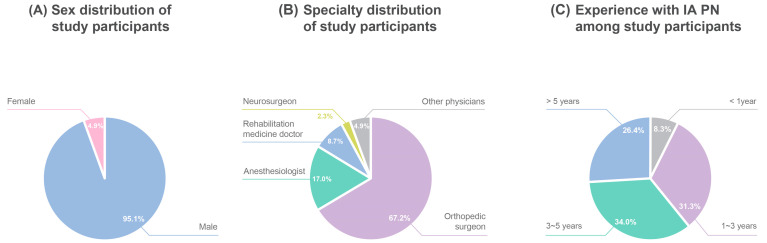
Characteristics of participants. (**A**): Sex distribution of study participants; (**B**): specialty distribution of study participants; (**C**): experience with IA PN among study participants. IA, intra-articular; PN, polynucleotide.

A total of 265 clinicians were included in the final analysis. The participants in this study were predominantly orthopedic surgeons (67.2%) and male (95.1%). The usage duration of PN among the clinicians was distributed as follows: 8.3% had been using PN for less than one year, 31.3% for 1–3 years, 34.0% for 3–5 years, and 26.4% for more than five years (Figure 1).

### 3.2. Current Practice Patterns

**Figure 2:**
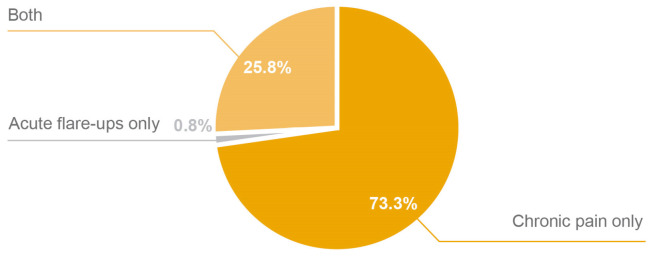
Clinician utilization of IA PN in knee osteoarthritis by pain type. IA, intra-articular; PN, polynucleotide.

**Figure 3:**
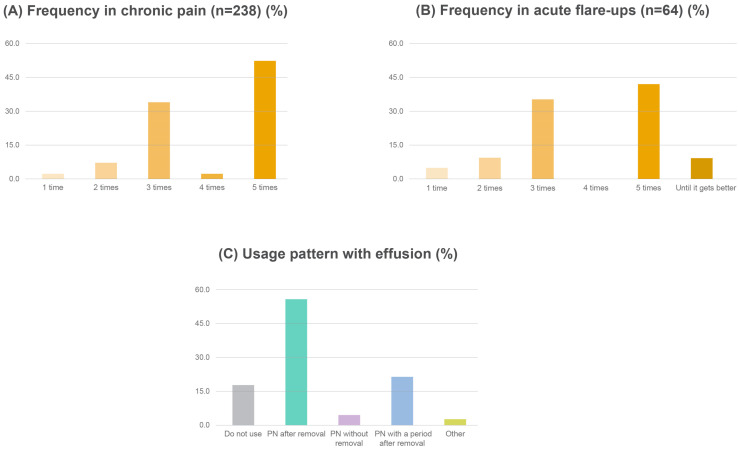
Clinician practices in IA PN for knee osteoarthritis. (**A**): Frequency for chronic pain; (**B**) frequency for acute flare-up; (**C**) approach in knee osteoarthritis with joint effusion. IA, intra-articular; PN, polynucleotide.

In terms of PN utilization in knee osteoarthritis, 73.3% (*n* = 176) of clinicians used PN only for treatment of chronic pain, 0.8% (*n* = 2) only for acute flare-ups, and 25.8% (*n* = 62) for both conditions (Figure 2). In cases of chronic pain (*n* = 238), the frequency of PN administration varied considerably. Specifically, 2.9% of clinicians administered PN once, 6.7% twice, 34.0% three times, 3.4% four times, and 52.9% five times (Figure 3A). Furthermore, clinicians (*n* = 64) used PN in acute flare-ups, with the majority administering PN three (34.4%) or five times (42.2%; Figure 3B). In the presence of effusion, the clinicians’ approaches were as follows: 17.4% of clinicians did not use PN, 55.5% removed the effusion before administering PN, 4.2% administered PN without removing the effusion, 20.4% removed the effusion and then waited before administering PN, and 2.6% had other approaches (Figure 3C).

### 3.3. Effectiveness

**Figure 4:**
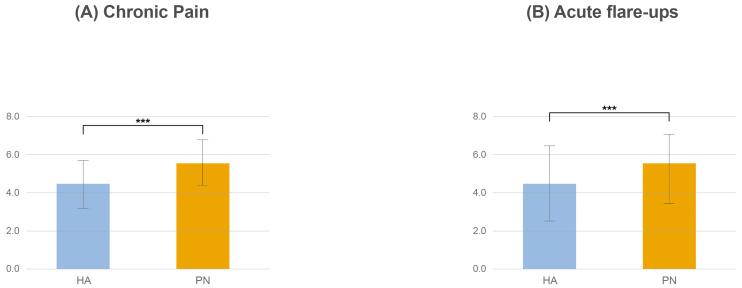
Comparative effectiveness of PN vs. HA by pain type. (**A**): Chronic pain; (**B**): acute flare-ups. PN, polynucleotide; HA, hyaluronic acid. *p* value: *** <0.001.

Effectiveness of PN compared with HA was evaluated for both chronic pain and acute knee osteoarthritis flare-up. For chronic pain, on a 10-point scale, PN had a higher average effectiveness score (7.0 ± 1.4) compared with HA (5.6 ± 1.5; *p*-value < 0.001; Figure 4A). For acute flare-up, PN also had a higher average effectiveness score (6.0 ± 2.3) compared with HA (4.9 ± 2.3; *p*-value < 0.001; Figure 4B).

**Figure 5:**
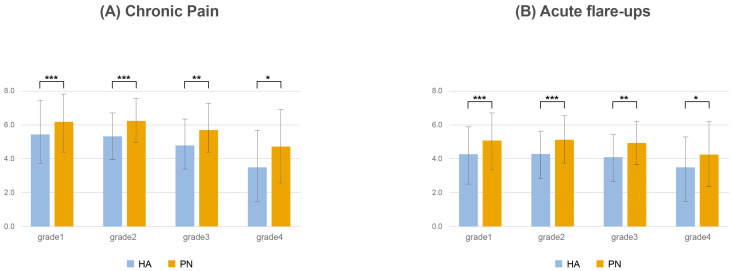
Comparative effectiveness of PN vs. HA across KL grades. (**A**): Chronic pain; (**B**): acute flare-ups. PN, polynucleotide; HA, hyaluronic acid. *p* value: * <0.05; ** <0.01; *** <0.001.

Clinicians consistently reported higher effectiveness for PN compared with HA across all Kellgren–Laurence (KL) grades in both chronic pain and acute flare-up. Notably, PN was perceived as more effective than HA for managing symptoms in both chronic pain and acute flare-up (Figure 5).

**Figure 6:**
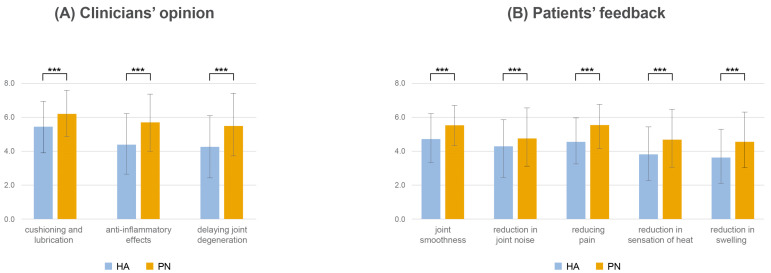
Comparative assessment of PN vs. HA: clinician and patient perspectives. (**A**) Clinicians’ opinion; (**B**) patients’ feedback. PN, polynucleotide; HA, hyaluronic acid. *p* value: *** <0.001.

The study evaluated the perceived effectiveness of HA and PN injections in the treatment of knee osteoarthritis as assessed by clinicians. The clinicians evaluated the effectiveness of HA and PN injections on a 10-point scale. The average score for IA HA in terms of cushioning and lubrication was 6.1 ± 1.7, while the score for IA PN was higher at 7.0 ± 1.6. Regarding anti-inflammatory effects, clinicians rated IA HA at 5.0 ± 2.0 and PN injections at 6.4 ± 2.0. In terms of delaying joint degradation, IA HA received a mean score of 4.8 ± 2.1 and IA PN a mean score of 6.2 ± 2.1 (Figure 6A).

Furthermore, patient feedback was rated. On average, joint smoothness had a score of 6.0 ± 1.7 with IA HA and 6.9 ± 1.6 with IA PN. A reduction in joint noise was observed with scores of 5.2 ± 2.1 for HA and 6.1 ± 2.2 for PN. Both treatments demonstrated efficacy in reducing pain, with HA achieving a mean score of 5.7 ± 1.7 and PN 6.9 ± 1.6. Furthermore, patients reported a reduction in sensation of heat (HA: 4.8 ± 2.0, PN: 5.9 ± 2.2) and swelling (HA: 4.6 ± 1.9, PN: 5.8 ± 2.1; Figure 6B).

## 4. Discussion

The survey showed that clinicians used PN injections based on clinical experience to manage both acute flare-ups and chronic pain in osteoarthritis. Most clinicians reported using five doses of PN for both uses. Clinicians perceived PN to be more effective than HA in chronic pain, noting that PN has superior anti-inflammatory and degeneration delaying effects. This contrasts with initial expectations that HA would perform better in terms of lubrication and cushioning. In addition, HA was expected to be preferred for the chronic management of osteoarthritis patients, and PN would be preferred over HA for acute flares. However, the survey of actual use showed that PN is preferred over HA for both chronic management and acute flares. PN was administered either immediately after effusion drainage or at intervals in acute cases.

While IA PN has shown promising results in the treatment of knee osteoarthritis, the potential complications associated with its use should not be overlooked. In previous studies, complications related to PN are relatively rare, but may include synovitis or inflammatory reactions in some cases. Compared with HA, PN injections appear to have a lower incidence of adverse effects such as pseudo-sepsis, often reported with HA injections. Nevertheless, further large-scale studies are needed to comprehensively evaluate the safety profile of PN, especially in the context of repeated use or combined therapies.

We observed significantly higher mean efficacy scores for PN than IA HA for both chronic pain and acute flare-up (*p* < 0.001 for both). When evaluating the effectiveness of HA and PN, clinicians rated the buffering/lubricating effect, anti-inflammatory effect, and degeneration delay higher for PN than for HA. Patient feedback showed higher scores for PN in all categories, including joint lubrication, noise reduction, pain reduction, heat sensation reduction, and swelling reduction.

When injected into the joint cavity, PN absorbs the surrounding moisture and takes on the viscoelastic properties of a gel. This physically restores the space between the joints and reduces mechanical friction, leading to the relief of knee joint pain [6,11,12]. In addition, evidence shows that certain DNA fractions may have a beneficial effect on tissue repair [14]. These fractions are thought to stimulate the synthesis of nucleic acids, which may contribute to the repair and regeneration of damaged tissue. Furthermore, these DNA fractions have been suggested to bind to purinergic receptors involved in various physiological processes and tissue repair [15].

Previous studies comparing PN with the widely used HA for knee osteoarthritis have demonstrated that pain in the group receiving three injections of PN improved more quickly and significantly compared to the group receiving three injections of HA [11]. This emphasizes the potential of PN as an alternative treatment option for knee osteoarthritis. In a previous in vivo study, it was observed that PN demonstrated anti-inflammatory effects in an OA cell model. This was achieved by partially inhibiting the NF-kB pathway and enhancing the Smad2/3 pathway, which resulted in a reduction in the expression of inflammatory markers (MMP3, MMP13, iNOS, COX-2) and an increase in extracellular matrix components such as aggrecan and collagen II [16].

The results of this survey showed that approximately one-quarter of physicians used PN for the treatment of acute flare-ups. The results of recent studies and clinical observations indicated that PN can effectively reduce inflammation, stabilizing vascular tissues and decreasing the levels of pro-inflammatory cytokines such as TNF-α [17]. This anti-inflammatory mechanism renders PN as a promising treatment for acute flare-ups, offering an alternative approach for reducing inflammation and promoting joint health.

Treatment options for knee osteoarthritis are limited, with most focusing on pain relief and functional impairment in daily life. Although IA injection therapy is widely used, many guidelines recommend only HA and corticosteroids. HA injections are controversial due to a lack of evidence of superiority over placebo [3], leading to downgraded recommendations from the American Academy of Orthopedic Surgeons [1] and the American College of Rheumatology [18]. However, the demand for these injections continues in clinical practice. Although the anti-inflammatory effects of IA HA and its potential to delay the progression of osteoarthritis are debatable, IA HA remains a recognized pharmacological treatment modality as well as NSAIDs. The results of clinical trials indicated that corticosteroid injections pose risks such as cartilage damage [19]. Consequently, there is a need for safe and effective treatment options for repeated IA injections. Therefore, it is important to compare the HA injection modality with more promising alternatives such as PN to provide better options for IA injection therapies.

To the best of our knowledge, this is the first study in which the current use patterns and perceived effectiveness of PN for knee osteoarthritis were investigated among clinicians. The survey included clinicians who used PN in real-world situations, and the results provide practical information to guide the use of PN and a basis for further research.

In the present study, a survey was conducted among clinicians to investigate the current practices and perceived effectiveness of PN to treat knee osteoarthritis. Although sufficient data were collected and broad coverage was provided, several limitations should be acknowledged. The participants were exclusively from the Republic of Korea, which may limit the generalizability of the findings. Additionally, the effectiveness of PN was evaluated solely based on surgeons’ perceptions using a 10-point scale, without incorporating standardized clinical or functional scores. The absence of specific patient demographic and clinical data, such as age or osteoarthritis grade, also limited the ability to stratify outcomes. A possibility of misunderstanding or biased responses also exists due to the wording of the questions. Despite these limitations, the results of this study provide a comprehensive overview of PN use in treating knee osteoarthritis. However, based on the promising results observed in clinical practice, further research is necessary to evaluate the effectiveness of PN in acute flare-ups, and studies involving patients with clinical data and standardized outcome measures are essential to validate these findings.

## 5. Conclusions

In summary, clinicians in the Republic of Korea have injected PN for the treatment of acute flare-ups or chronic pain. These findings support the hypothesis that PN can be beneficial for knee osteoarthritis treatment.

## Data Availability

The data that support the findings of this study are available upon reasonable request from the corresponding author.
